# Hydrogen Indirectly Suppresses Increases in Hydrogen Peroxide in Cytoplasmic Hydroxyl Radical-Induced Cells and Suppresses Cellular Senescence

**DOI:** 10.3390/ijms20020456

**Published:** 2019-01-21

**Authors:** Takahiro Sakai, Ryosuke Kurokawa, Shin-ichi Hirano, Jun Imai

**Affiliations:** 1 Laboratory of Physiological Chemistry, Faculty of Pharmacy, Takasaki University of Health and Welfare, 60 Nakaorui-machi, Takasaki, Gunma 370-0033, Japan; jimai@takasaki-u.ac.jp; 2 MiZ Co., Ltd., 2-19-15 Ofuna, Kamakura, Kanagawa 247-0056, Japan; r_kurokawa@e-miz.co.jp (R.K.); s_hirano@e-miz.co.jp (S.-i.H.)

**Keywords:** cytoplasmic hydroxyl radical, hydrogen, hydrogen peroxide, lipid peroxide, cellular senescence

## Abstract

Bacteria inhabiting the human gut metabolize microbiota-accessible carbohydrates (MAC) contained in plant fibers and subsequently release metabolic products. Gut bacteria produce hydrogen (H_2_), which scavenges the hydroxyl radical (•OH). Because H_2_ diffuses within the cell, it is hypothesized that H_2_ scavenges cytoplasmic •OH (cyto •OH) and suppresses cellular senescence. However, the mechanisms of cyto •OH-induced cellular senescence and the physiological role of gut bacteria-secreted H_2_ have not been elucidated. Based on the pyocyanin-stimulated cyto •OH-induced cellular senescence model, the mechanism by which cyto •OH causes cellular senescence was investigated by adding a supersaturated concentration of H_2_ into the cell culture medium. Cyto •OH-generated lipid peroxide caused glutathione (GSH) and heme shortage, increased hydrogen peroxide (H_2_O_2_), and induced cellular senescence via the phosphorylation of ataxia telangiectasia mutated kinase serine 1981 (p-ATM^ser1981^)/p53 serine 15 (p-p53^ser15^)/p21 and phosphorylation of heme-regulated inhibitor (p-HRI)/phospho-eukaryotic translation initiation factor 2 subunit alpha serine 51 (p-eIF2α)/activating transcription factor 4 (ATF4)/p16 pathways. Further, H_2_ suppressed increased H_2_O_2_ by suppressing cyto •OH-mediated lipid peroxide formation and cellular senescence induction via two pathways. H_2_ produced by gut bacteria diffuses throughout the body to scavenge cyto •OH in cells. Therefore, it is highly likely that gut bacteria-produced H_2_ is involved in intracellular maintenance of the redox state, thereby suppressing cellular senescence and individual aging. Hence, H_2_ produced by intestinal bacteria may be involved in the suppression of aging.

## 1. Introduction

Intestinal bacteria, which inhabit the human gut, metabolize rich stores of microbiota-accessible carbohydrates (MAC) contained in plant fibers, releasing various metabolic products that play important roles in the host’s immune functions, metabolism, and homeostatic maintenance. However, while many of these gut bacteria produce H_2_, the physiological role of H_2_ in vivo is not yet understood. Recent research has shown that H_2_ specifically neutralizes hydroxyl radicals (•OH), which have the strongest oxidizing power among reactive oxygen species (ROS) [1]. Recently, it was suggested that •OH located in the cytoplasm (cyto •OH), unlike •OH localized in the mitochondria (mito •OH), is one of the inducers of cellular senescence [2]. Senescent cells accumulate in aged tissues, and elimination of senescent cells suppresses tissue and organ dysfunction [3]. In fact, suppression of cellular senescence has been suggested to delay individual aging and prolong a healthy lifespan [4]. Accordingly, we hypothesized that the H_2_ produced by gut bacteria circulates throughout the host’s body, delaying aging by suppressing cellular senescence and extending the host’s healthy lifespan. Because H_2_ can diffuse into cells, H_2_ is expected to scavenge cyto •OH and suppress cellular senescence. However, there is currently a lack of evidence to support this. Thus, the physiological role of H_2_ produced by gut bacteria and the mechanism by which cyto •OH causes cellular senescence remain unclear. In this study, we clarified the induction mechanism of cellular senescence caused by cyto •OH by adding a solution of supersaturated H_2_ to the cyto •OH-induced cellular senescence model stimulated by pyocyanin, which is a cytoplasmic ROS inducer in vitro. Because cyto •OH specifically produces lipid peroxide, we assumed that lipid peroxide is involved in the induction of cellular senescence. Therefore, we speculated that cyto •OH scavenging by intestinal bacteria-produced H_2_ would suppress lipid peroxide production and regulate cellular and individual aging.

## 2. Results

### 2.1. H_2_ Suppressed p16 and p21 Expression and Cellular Senescence

**Figure 1:**
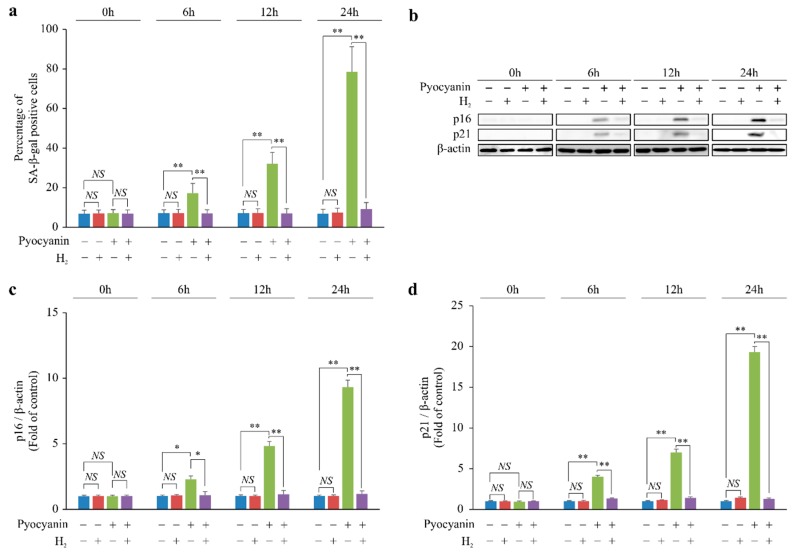
H_2_ suppressed cytoplasmic hydroxyl radical (cyto •OH)-induced cellular senescence via p16 and p21 in vitro. (**a**–**d**) The effect of H_2_ at 0, 6, 12, and 24 h in the cyto •OH-induced oxidative stress model using pyocyanin-stimulated mouse embryonic fibroblasts (MEFs). (**a**) Cellular senescence and (**b**,**c**) p16 and (**b**,**d**) p21 expression in pyocyanin-stimulated MEFs determined by β-galactosidase (SA-β-gal) staining and Western blot analysis, respectively. The data are presented as the mean ± SEM (*n* = 5 per group); * *p* < 0.05; ** *p* < 0.01; NS, not significant.

We stimulated mouse embryonic fibroblasts (MEFs) with the cyto •OH inducer pyocyanin and examined the effect of H_2_ on cellular senescence. Although pyocyanin increased cellular senescence chronologically, H_2_ suppressed this effect (Figure 1a). Furthermore, we examined the effect of H_2_ on the expression of cellular senescence regulators p16Ink4a (p16) and p21Cip1/Waf1 (p21). Chronologically, pyocyanin increased the expression of p16 and p21, which was suppressed by H_2_ (Figure 1b–d). Overall, these findings showed that H_2_ suppressed p21 and p16 expression and cellular senescence induced by cyto •OH.

### 2.2. H_2_ Indirectly Suppressed Heme Depletion and Cellular Senescence via the p-HRI/p-eIF2α/ATF4/p16 Pathway

**Figure 2:**
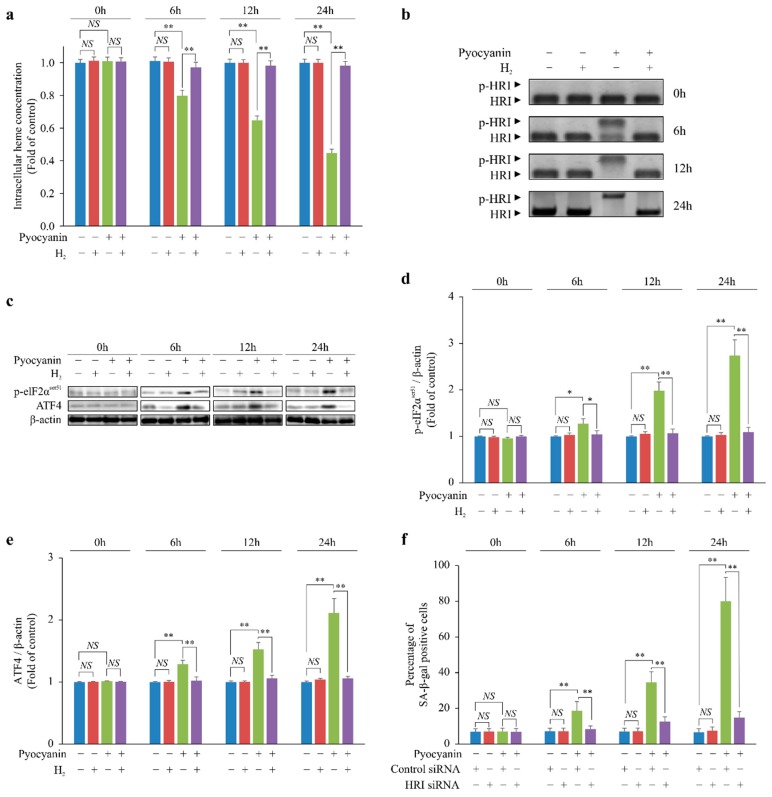
Scavenging of cyto •OH by H_2_ suppressed heme depletion and induced p16 expression in pyocyanin-stimulated MEFs. (**a**) Intracellular heme concentration. Phosphorylation of (**b**) heme-regulated inhibitor (HRI) and (**c**,**d**) eIF2α^ser51^ and expression of (**c**,**e**) activating transcription factor 4 (ATF4) based on Western blot analysis. (**f**) Cellular senescence in pyocyanin-stimulated MEFs after knockdown of *HRI* mRNA determined by SA-β-gal staining. The data are presented as the mean ± SEM (*n* = 5 per group); * *p* < 0.05; ** *p* < 0.01; NS, not significant.

p16 is induced by phospho-eukaryotic translation initiation factor 2 subunit alpha serine 51 (p-eIF2α^ser51^)–mediated activation of activating transcription factor 4 (ATF4) [5,6]. eIF2α is activated after phosphorylation by a heme-regulated inhibitor (HRI) [7], which is phosphorylated through heme depletion [8]. We investigated the chronological changes in the intracellular heme concentration of pyocyanin-stimulated MEFs. Pyocyanin chronologically decreased the intracellular heme concentration, but H_2_ suppressed this decrease, similar to the p16 expression pattern (Figure 2a). Next, we examined phosphorylation of HRI (p-HRI) and eIF2α^ser5^ and ATF4 expression. Pyocyanin chronologically increased p-HRI and eIF2α^ser5^ and ATF4 expression (Figure 2b–e). In contrast, H_2_ suppressed p-HRI and eIF2α^ser5^ and ATF4 expression, similar to the pattern of changes in intracellular heme concentration (Figure 2b–e). Furthermore, when HRI was knocked down by RNA interference, cellular senescence caused by pyocyanin stimulation was suppressed (Figure 2f). 

Together, the results suggested that H_2_ suppressed cellular senescence via the p-HRI/p-eIF2α^ser5^/ATF4/p16 pathway by suppressing heme depletion. 

### 2.3. H_2_ Suppressed Lipid Peroxide Production and Increased H_2_O_2_ by Suppressing Glutathione (GSH) Depletion

**Figure 3:**
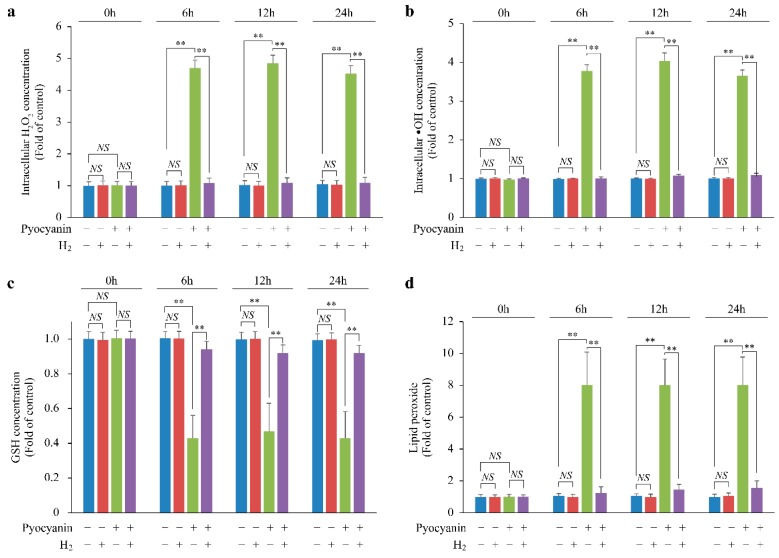
Scavenging of cyto •OH by H_2_ suppressed lipid peroxide production and glutathione (GSH) depletion and indirectly maintained the increase in H_2_O_2_. (**a**) H_2_O_2_, (**b**) cyto •OH, (**c**) GSH, and (**d**) lipid peroxide concentrations in pyocyanin-stimulated MEFs. The data are presented as the mean ± SEM (*n* = 5 per group); ** *p* < 0.01; NS, not significant.

Pyocyanin markedly increased the chronological intracellular H_2_O_2_ and •OH concentration, which was inhibited by H_2_ (Figure 3a,b). In addition, pyocyanin reduced GSH, one of the H_2_O_2_ scavengers, but H_2_ suppressed the GSH decrease (Figure 3c). Moreover, pyocyanin enhanced lipid peroxide production, but this was suppressed by H_2_ (Figure 3d). 

Overall, these data suggest that H_2_ suppressed the increase in lipid peroxide produced by •OH, suppressed GSH depletion, and indirectly suppressed the increase in H_2_O_2_. 

### 2.4. H_2_ Indirectly Suppressed Intracellular H_2_O_2_ Concentration Increase and DNA Oxidative Damage and Suppressed Cellular Senescence via p-ATM^ser1981^/p-p53^ser15^/p21 Pathway

**Figure 4:**
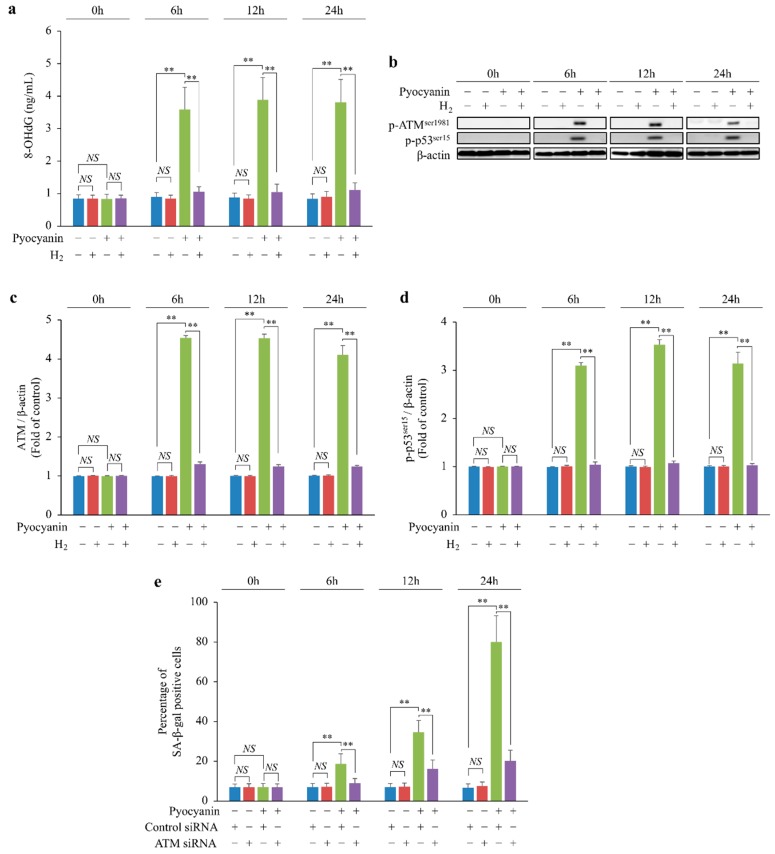
Scavenging of cyto •OH by H_2_ suppressed cellular senescence via the ATM/p-p53^ser15^ pathway by suppressing the intracellular H_2_O_2_ concentration increase and DNA damage. (**a**) DNA oxidative damage detected by marker 8-hydroxy-2-deoxyguanosine (8-OHdG); (**b**–**d**) phosphorylation of (**b**,**c**) ATM^ser1981^ and (**b**,**d**) p53^ser15^ detected by Western blot analysis; (**e**) cellular senescence in pyocyanin-stimulated MEFs following knockdown of *ATM* mRNA determined by SA-β-gal staining. The data are presented as the mean ± SEM (*n* = 5 per group); ** *p* < 0.01; NS, not significant.

We examined the chronological DNA oxidative damage in MEFs stimulated with pyocyanin. Pyocyanin increased chronological DNA oxidative damage in MEFs, but H_2_ suppressed this damage (Figure 4a). In addition, we examined the phosphorylation of ataxia telangiectasia mutated kinase serine 1981 (p-ATM^ser1981^) and p53 serine 15 (p-p53^ser15^) induced by DNA oxidative damage. Pyocyanin increased the phosphorylation of ATM^ser1981^ and p53^ser15^ in MEFs, but H_2_ suppressed these reactions (Figure 4b–d). Furthermore, when ATM was knocked down by RNA interference, cellular senescence caused by pyocyanin stimulation was suppressed (Figure 4e). 

Together, these data indicate that H_2_ indirectly suppressed increases in intracellular H_2_O_2_ concentration and oxidative DNA damage and suppressed cellular senescence via the p-ATM^ser1981^/p-p53^ser15^/p21 pathway.

## 3. Discussion

Several gut bacteria are known to produce H_2_; however, the physiological role of this H_2_ and the mechanism by which cyto •OH causes cellular senescence have not been elucidated. Here, the induction mechanism of cellular senescence caused by cyto •OH was investigated.

•OH can be generated by ferric ions without any oxidizing agent through the Fenton reaction. In view of the well-known damaging effect of poorly chelated iron on the human body, numerous natural products containing iron-binding agents or •OH scavengers may be essential for the maintenance of human health [9]. However, intestinal bacteria produce H_2_ that specifically scavenges •OH [10]. In recent studies using H_2_ gas and H_2_ water, H_2_ was found to be useful for the treatment of various geriatric diseases involving oxidative stress [1]. In a previous study using cyto •OH scavenger TA293, we demonstrated that cyto •OH induced cellular senescence in vitro and in vivo [2]. In this study, similar to TA293, we found that H_2_ suppressed the cellular senescence of pyocyanin-stimulated cells. 

**Figure 5:**
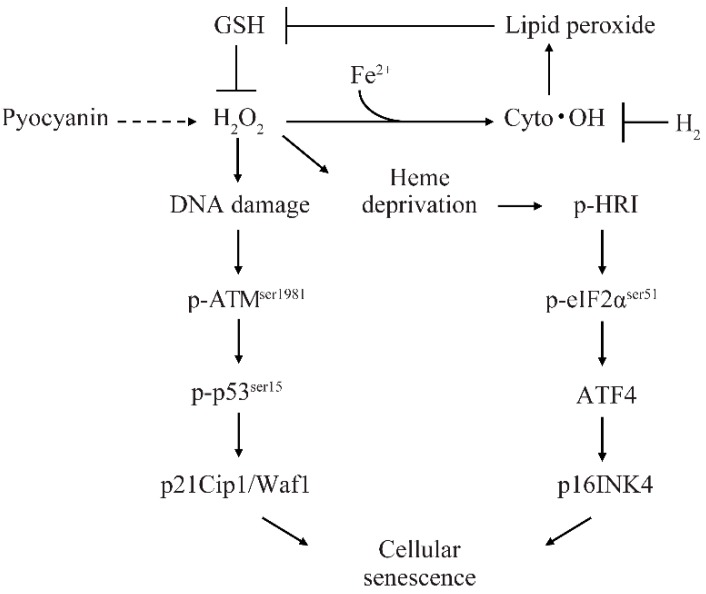
Schematic diagram of cyto •OH-induced cellular senescence model. The solid arrows indicate direct induction, the dotted arrows indicate indirect induction, and the “T” arrows indicate inhibition.

Recently, cellular senescence has been implicated as a direct cause of the age-related phenotype [4]. However, the mechanism of cyto •OH-induced cellular senescence is unknown. We propose a cyto •OH-induced cellular senescence model involving H_2_ (Figure 5), in which GSH inhibits H_2_O_2_, cyto •OH produces lipid peroxide, and these markedly decrease intracellular GSH. The depletion of GSH cannot sufficiently inhibit H_2_O_2_, and excess H_2_O_2_ causes oxidative DNA damage and induces p21 expression via the p-ATM^ser1981^/p-p53^ser15^ pathway. Furthermore, lipid peroxide reacts readily with transition metal ions, such as copper and iron, and heme protein to generate reactive carbonyls [11]. In addition, heme proteins that reduce H_2_O_2_, such as GSH peroxidase and catalase, contain a heme prosthetic group [12]. Based on these findings, we expect that either lipid peroxide directly reduces heme or production of excessive H_2_O_2_ by these antioxidant enzymes causes intracellular heme deficiency. HRI is thought to recognize intracellular heme deficiency and induce p16 expression via the p-HRI/p-eIF2α^ser51^/ATF4 pathway. 

In contrast, H_2_ suppresses lipid peroxide production and prevents GSH peroxidase activation and GSH depletion. By preventing GSH depletion, H_2_O_2_ is stably eliminated by GSH, and excessive catalase activation is suppressed. Reduction in overproduced H_2_O_2_ suppresses DNA oxidative damage and induction of the p-ATM^ser1981^/p-p53^ser15^/p21 pathway. Similar to GSH peroxidase and catalase, suppression of heme protein activation prevents heme depletion and suppresses p16 activation via the p-HRI/p-eIF2α^ser51^/ATF4 pathway. 

In this study, we found that scavenging of cyto •OH by H_2_ indirectly suppressed the increase of H_2_O_2_ and suppressed cellular senescence in a cyto •OH induced cellular senescence model. H_2_ has been known to suppress cellular senescence as well as the expression of p16 and p21 in other ROS-induced cellular senescence models, which supports the findings of our study [13,14,15,16]. H_2_ reduced the levels of other intracellular ROS despite being an •OH specific scavenger [16,17]. 

In this study, we suggested that cyto •OH is an inducer causing cellular senescence. However, it is thought that mito •OH is not an inducer of cellular senescence, as the decrease in mitochondrial localized superoxide dismutase 2 (SOD2) activity induces apoptosis but not cellular senescence [18,19]. On the other hand, since H_2_ diffuses within the cell, it scavenges not only cyto •OH but also mito •OH [1]. TNFα and IL-1β activate SOD2 and produce H_2_O_2_. [18,20]. H_2_O_2_ produced by the activation of SOD2 increases the oxidation of mitochondrial DNA (mtDNA) and the peroxidation of the mitochondrial membrane [19]. Oxidized mtDNA then activates the NLRP3 inflammasome and induces secretion of caspase-1, IL-8, and IL-1β [21]. Peroxidation of the mitochondrial membrane releases cytochrome *c* and induces apoptosis [22]. Therefore, we believe that H_2_ is more likely to suppress apoptosis and the secretion of inflammatory cytokines, in addition to suppressing cellular senescence.

One limitation of this study was that we did not confirm whether the H_2_ produced by intestinal bacteria suppresses cellular senescence and individual aging in vivo. Nevertheless, many previous in vivo observations support our hypothesis, such as the H_2_-induced suppression of cognitive memory disorder and degeneration of hippocampal neurons in senescence-accelerated mouse-prone 8 (SAMP8) mice [23]. In addition, suppressed cell death of dopaminergic neurons in a Parkinson’s disease animal model [24,25], prevention of atherosclerosis in an apolipoprotein E knockout (apoE KO) mouse [26], and suppressed mild cognitive impairment in a dementia animal model [27] provide supporting evidence for in vivo effects. Cyto •OH scavenging is also known to suppress cellular senescence and inflammation [2]. Moreover, NO, O_2_^−^, and the •OH scavenger phloroglucinol suppress H_2_O_2_-induced premature senescence and inhibit lipid peroxidation [28]. Furthermore, through a study of mice [13], it was determined that ingesting H_2_ water can effectively suppress cellular senescence and individual aging. Our findings suggest that H_2_ is a promising molecule for suppression of cellular senescence-related geriatric diseases and may have an implication in the control of geriatric diseases. Therefore, increasing H_2_ production by intestinal bacteria using MAC is highly likely to suppress cyto •OH-induced cellular senescence and inhibit individual aging.

## 4. Materials and Methods

### 4.1. Reagents

Pyocyanin was purchased from Cayman Chemical (San Diego, CA, USA).

### 4.2. Production of H_2_ Super-Rich Medium and Measurement of H_2_ Content in Medium

We used Dulbecco’s modified Eagle’s medium (DMEM) without phenol red purchased from Thermo Fisher Scientific Inc (Waltham, MA, USA). A solution of high-concentration, H_2_-rich medium (7.0 ppm) was prepared using an H_2_-generating agent (MiZ Co. Ltd., Kanagawa, Japan) as described previously [29]. The concentration of dissolved H_2_ in DMEM without phenol red was 7.0 ppm, as measured using a dissolved H_2_ reagent methylene blue kit (MiZ Co. Ltd., Kanagawa, Japan) [30]. 

### 4.3. Primary Mouse Embryonic Fibroblast (MEF) Cell Culture and ROS Induction

MEFs were obtained from E13.5 embryos of C57BL/6J mice (purchased from CLEA, Inc., Tokyo, Japan), using the standard protocol [31,32]. MEFs were treated with vehicle or ROS inducer (30 μM pyocyanin) in normal medium or H_2_ super-rich DMEM. In this study, MEFs were used at different passages (P1–10). All animal experiments conducted during this study strictly adhered to the Guide for the Care and Use of Laboratory Animals by the US National Institutes of Health (NIH Publications No. 8023, revised 1978). All experimental procedures were approved by the animal experiment ethics committee of our university and by the animal experiment ethics committee of the Takasaki University on Health and Welfare (Certificate Number: 1814, Approval date: 01 April 2018).

### 4.4. Measurement of Senescence-Associated β-galactosidase (SA-β-gal) Activity

Senescent cells induced by pyocyanin stimulation were assessed by SA-β-gal staining using the Senescence Detection Kit (BioVision, Mountain View, CA, USA) following the manufacturer’s instructions. Briefly, the cells were fixed in 2% formaldehyde and incubated with staining solution for 16 h at 37 °C. Subsequently, the cells were washed with PBS and mounted with Permount (Fisher Scientific, Fair Lawn, NJ, USA). Finally, positively stained cells were counted under a microscope at 20× magnification in five random fields for each experimental condition.

### 4.5. Western Blotting

MEFs were homogenized on ice in RIPA buffer (20 mM Tris-HCl (pH 7.4), 150 mM NaCl, 1% NP-40, 1% sodium deoxycholate, and 0.1% SDS) containing protease and phosphatase inhibitors (cOmplete Mini and phosSTOP, respectively; Roche Diagnostics GmbH, Mannheim, Germany) and centrifuged at 11,000× *g* for 30 min. The supernatant protein concentrations were estimated using the Bradford assay (Bio-Rad, Hercules, CA, USA). 

Western blotting was conducted following the same procedures described in a previous study [2] using primary antibodies against p16Ink4a (Bioworld Technology, St. Louis Park, MN, USA), p21Waf1/Cip1 (Santa Cruz Biotechnology, Dallas, TX, USA), ATF4, p-p53^ser15^, p-eIF2α^ser51^, β-actin (Cell Signaling Technology, Beverly, MA, USA), p-ATM^ser1981^ (Rockland Immunochemicals Inc., Limerick, PA, USA), and HRI (Millipore, Sydney, Australia) diluted 1:1000. After incubation with horseradish peroxidase (HRP)-linked secondary antibodies diluted 1:2000 (Cell Signaling Technology), signals were detected using ECL Plus Western Blotting Detection Reagents (GE Healthcare Life Sciences, Marlborough, MA, USA). Quantification of bands was performed by densitometry using Multi Gauge ver. 3.0 software (FUJIFILM Corp., Tokyo, Japan). 

### 4.6. Quantitation of Intracellular Heme

Intracellular heme was quantified in MEFs using a heme assay kit (Sigma-Aldrich, St. Louis, MO, USA) according to the manufacturer’s instructions. Briefly, 200 μL of heme reagent was added to 50 μL of sample. Subsequently, the samples were vortexed to mix. The plate containing the mixtures was incubated for 5 min at room temperature (about 22 °C), and absorbance was measured at 400 nm.

### 4.7. Measurement of Lipid Peroxide

Lipid peroxide was quantified using the lipid peroxide fluorescent probe SPY-LHP (Dojindo Laboratories, Kumamoto, Japan) according to the manufacturer’s instructions. Briefly, MEFs were incubated with 100 μM SPY-LHP in medium for 30 min at 37 °C. Subsequently, the cells were washed twice in PBS and then immediately analyzed using a Wallac 1420 ARVO MX multilabel counter (PerkinElmer Co., Ltd., Waltham, MA, USA).

### 4.8. Knockdown of HRI and ATM in Cells

siRNA transfections were performed using Lipofectamine RNAiMAX reagent (Life Technologies, Carlsbad, CA, USA) according to the manufacturer’s instructions. HRI- and ATM-specific siRNAs and non-targeting siRNA negative controls were purchased from Santa Cruz Biotechnology.

### 4.9. Measurement of Intracellular H_2_O_2_

Quantitation of H_2_O_2_ was performed using the OxiSelect™ Hydrogen Peroxide/Peroxidase Assay Kit (Cell Biolabs, Inc., San Diego, CA, USA) according to the manufacturer’s instructions. Briefly, 50 µL (1 × 10^6^ cells) of MEFs prepared in assay buffer and 50 µL of H_2_O_2_ working solution were mixed and incubated for 30 min at room temperature, protected from light. Absorbance was then measured at 540 nm to quantify intracellular H_2_O_2_ in cells.

### 4.10. Measurement of Oxidative DNA Damage

Oxidative DNA damage in MEFs was evaluated by first extracting the DNA using the DNA Extractor TIS Kit (JaICA, Shizuoka, Japan) according to the manufacturer’s instructions. DNA damage was assessed by measuring 8-hydroxy-2-deoxyguanosine (8-OHdG) using the 8-OHdG EIA kit (JaICA) according to the manufacturer’s instructions, as previously described [2].

### 4.11. In Vitro Electron Spin Resonance (ESR) Measurements

After centrifugation at 280× *g* for 5 min to remove the supernatant, MEFs were pre-incubated in 0.3 mL PBS containing 0.1 M 5,5-dimethyl-1-pyrroline N-oxide (DMPO) for 7 min at 20 °C. Subsequently, the mixture was transferred to a glass capillary for ESR experiments. These samples were measured using an ESR JES-REIX X-band spectrometer (JEOL, Tokyo, Japan). The •OH detection conditions were as follows: field, 336 ± 5 mT width; power, 4 mW; field modulation, 0.200 mT; time constant, 0.1 s; and amplitude, 500. A manganese signal was used for the external standard.

### 4.12. Quantitation of Intracellular GSH

Intracellular GSH was determined with a quantification kit (Dojindo Laboratories) according to the manufacturer’s instructions. Briefly, MEFs were lysed by the addition of 80 μL of 10 mM HCl and three freeze-thaw cycles. To the homogenate, 20 μL of 5% salicylsalicylic acid (SSA) was added, and the mixture was centrifuged at 8000× *g* for 10 min. GSH levels in the supernatant were determined according to the manufacturer’s protocol by measuring absorbance at 405 nm with a Sunrise absorbance microtiter plate reader (Tecan Japan, Kanagawa, Japan).

### 4.13. Statistical Analysis

The data are presented as the mean ± standard error of the mean (SEM) based on five independent experiments. Statistical analysis was performed by one-way analysis of variance (ANOVA) using Excel Statics (BellCurve, Tokyo, Japan) and IBM SPSS Statistics (Tokyo, Japan), and *p* < 0.05 was considered statistically significant. 

## 5. Conclusions

Our study suggests a model whereby the lipid peroxide produced by cyto •OH causes a decrease in GSH and heme storage, increases H_2_O_2_, and induces cellular senescence through the oxidative DNA damage/p-ATM^ser1981^/p-p53^ser15^/p21 pathway and heme depletion/p-HRI/p-eIF2α^ser51^/ATF4/p16 pathway. Our findings also indicate that H_2_ is a promising molecule for the suppression of geriatric disease. Therefore, the relationship between H_2_-secreting intestinal bacteria and geriatric diseases should be further investigated.

## Abbreviations

8-OHdG	8-Hydroxy-2-deoxyguanosine
ATF4	Activating transcription factor 4
ATM	Ataxia telangiectasia mutated kinase
cyto •OH	Cytoplasmic hydroxyl radical
DMEM	Dulbecco’s modified Eagle’s medium
ESR	Electron spin resonance
GSH	Glutathione
HRI	Heme-regulated inhibitor
MAC	Microbiota-accessible carbohydrates
MEF	Mouse embryonic fibroblast
•OH	Hydroxyl radical
p-eIF2α^ser51^	Phospho-eukaryotic translation initiation factor 2 subunit alpha serine 51
ROS	Reactive oxygen species
SA-β-gal	Senescence-associated β-galactosidase
